# 

**Published:** 1848

**Authors:** 


					﻿WHOLE SERIES----VOL. V. NO. II.
Vol. I.]
JUNE AND JULY.
[No. 2.
THE NORTH-WESTERN
MEDICAL AND SURGICAL
JOURNAL.
z
o
iS
Pä
g
P
o
£
g
a
a
g
co
J
M
0
3
O
ö
o
f
r
>
w
co
S
PS
>
1
s
2
>
ü
>
2J.
Q
M
EDITED BY
WILLIAM B. HERRICK, M.D., AND JOHN EVANS, M.D.,
PROFESSORS IN RUSH MEDICAL COLLEGE, CHICAGO.
CHICAGO AND INDIANAPOLIS:
PUBLISHED BY JOHN W. DUZAN.
INDIANA STATE JOURNAL—PRINT.
1 8 4 8.
ENLARGED SERIES—VOE. Ill, NO. II.
				

